# Anti-TIGIT differentially affects sepsis survival in immunologically experienced versus previously naive hosts

**DOI:** 10.1172/jci.insight.141245

**Published:** 2021-03-08

**Authors:** Yini Sun, Jerome C. Anyalebechi, He Sun, Tetsuya Yumoto, Ming Xue, Danya Liu, Zhe Liang, Craig M. Coopersmith, Mandy L. Ford

**Affiliations:** 1 Department of Surgery, Emory University School of Medicine, Atlanta, Georgia, USA.; 2 Department of Critical Care Medicine, The First Affiliated Hospital of China Medical University, China Medical University, Shenyang, China.; 3 Emory Critical Care Center, Emory University School of Medicine, Atlanta, Georgia, USA.; 4 Department of Hepatobiliary Surgery and Transplantation, The First Affiliated Hospital of China Medical University, China Medical University, Shenyang, China.; 5 Emory Transplant Center, Emory University School of Medicine, Atlanta, Georgia, USA.; 6 Department of Critical Care Medicine, Zhongda Hospital, School of Medicine, Southeast University, Nanjing, China.

**Keywords:** Infectious disease, Inflammation, Apoptosis, Costimulation, T cells

## Abstract

Mounting evidence suggests that the balance of T cell costimulatory and coinhibitory signals contributes to mortality during sepsis. Here, we identified a critical role of the coinhibitory molecule T cell Ig and ITIM domain (TIGIT) in regulating sepsis mortality. Because TIGIT is significantly upregulated on memory T cells, we developed a “memory mouse” model to study the role of TIGIT during sepsis in a more physiologically relevant context. Mice received sequential pathogen exposure and developed memory T cell frequencies, similar to those observed in adult humans, and were then subjected to sepsis induction via cecal ligation and puncture. Our results show that targeting the TIGIT pathway during sepsis is fundamentally different in previously naive versus memory mice, in that αTIGIT Ab had no effect on survival in previously naive septic mice but sharply worsened survival in memory septic mice. Mechanistically, αTIGIT increased apoptosis of memory T cells, decreased T cell function, and downregulated the costimulatory receptor DNAM on memory CD8^+^ T cells in memory septic mice, but not in previously naive septic mice. Additionally, αTIGIT diminished Helios expression in Tregs in memory but not previously naive septic mice. These data highlight fundamental differences in the pathophysiological impact of targeting TIGIT in immunologically experienced versus previously naive hosts during sepsis.

## Introduction

Sepsis is a life-threatening organ dysfunction that results from a dysregulated host response to infection (1). It is the most common cause of death in the intensive care unit (ICU), resulting in 20% of ICU deaths worldwide (2). Although sepsis has been historically considered to be the result of an overwhelming inflammatory response, emerging evidence has demonstrated that disruption in the balance of costimulatory versus coinhibitory signaling contributes to increased morbidity and mortality during sepsis (3).

Accumulating evidence also suggests that apoptosis and dysfunction of T cells contributes to sepsis-induced immunosuppression (4). Additionally, analysis of samples from patients who died of sepsis revealed decreased T cell numbers, decreased T cell cytokine production, and increased T cell coinhibitory receptor expression (5). Specifically, T cell coinhibitory receptors PD-1, CTLA-4, and 2B4 have been shown to be highly expressed in septic mice and humans, and are being targeted clinically to improve antiseptic responses (6). Anti–PD-1/PD-L1 mAbs have been reported to improve the survival in experimental models of sepsis, and recent clinical trials are beginning to report positive outcomes (7, 8). Blockade of CTLA-4 also resulted in increased survival of the murine cecal ligation and puncture (CLP) model (9). Moreover, we recently demonstrated that blockade of the coinhibitory molecule 2B4 resulted in increased CLP survival in both previously healthy septic animals and in septic mice with preexisting malignancy (10, 11). Taken together, these studies demonstrate the potential therapeutic utility of checkpoint blockade in sepsis.

T cell Ig and ITIM domain (TIGIT) is a novel coinhibitory molecule that is expressed on activated human T cells and contains an Ig variable domain, a transmembrane domain, and an ITIM (12). TIGIT is mainly expressed on memory T cells, Tregs, as well as NK cells, and is upregulated on naive T cells upon activation. Similar to the CTLA-4/CD28 pathway, TIGIT competes with the costimulatory receptor CD226 (also known as DNAM) for the same set of ligands (CD155 and CD112) and mediates immune suppression in tumors and chronic infections (13–15). Our previous study showed that TIGIT expression on T and NK cells was upregulated in the mouse model of sepsis (our unpublished observations).

It is also well known that TIGIT expression is significantly altered on memory T cells compared with naive T cells in humans and mice (12). However, laboratory mice possess fewer memory T cells than adult humans or feral mice (16). Therefore, to better mimic the immune features of adults and explore the role of TIGIT in sepsis, we developed a “memory mouse” model, in which mice receive sequential pathogen exposure and possess frequencies of memory T cells similar to those observed in adult humans, followed by sepsis induction via CLP (17). We then interrogated the role of TIGIT signaling on memory T cells and explored the differential effects of αTIGIT on memory versus previously naive mice during sepsis. Results indicated that whereas αTIGIT had no effect on sepsis survival in previously naive septic mice, it resulted in significantly decreased survival in memory septic mice. Moreover, αTIGIT mAb exhibited markedly different effects on T cell apoptosis, cytokine effector function, and Treg stability in previously naive versus immunologically experienced hosts during sepsis.

## Results

### TIGIT expression on both CD4^+^ and CD8^+^ T cells is upregulated in memory mice relative to naive mice.

Multiple studies have shown that in addition to Tregs, TIGIT is primarily expressed on memory T cells and is absent on naive T cells (12, 14). To study TIGIT biology in a more physiologically relevant context, we developed a memory mice model via sequential infection of naive B6 mice with *Listeria monocytogenes* followed by lymphocytic choriomeningitis virus (LCMV) (Supplemental Figure 1A; supplemental material available online with this article; https://doi.org/10.1172/jci.insight.141245DS1). At 56 days after first infection, these antigen-experienced memory mice possessed approximately 23% memory CD44^hi^CD4^+^ T cells and 66% CD44^hi^CD8^+^ T cells in the blood, which is similar to the frequencies observed in adult humans (Supplemental Figure 1B) (16). In contrast, CD4^+^ and CD8^+^ T cell compartments of age-matched naive mice comprised only 15% and 19% memory T cells, respectively (Supplemental Figure 1, C and D). Additionally, the absolute numbers of memory CD4^+^ and CD8^+^ T cells in memory mice were also dramatically higher than those in naive mice (CD44^hi^CD4^+^, *P* = 0.0007, Supplemental Figure 1E; CD44^hi^CD8^+^, *P* = 0.0007, Supplemental Figure 1F).

To determine TIGIT expression on T cells, splenocytes from naive and memory mice were analyzed by flow cytometry (Supplemental Figure 1G). TIGIT expression was significantly increased on bulk CD4^+^ and CD8^+^ T cells in memory mice compared with naive mice (CD4: 5.4% ± 0.3% vs. 7.4% ± 0.5%, *P* = 0.0037, Supplemental Figure 1H; CD8: 1.7% ± 0.3% vs. 2.9% ± 0.3%, *P* = 0.04, Supplemental Figure 1I). Compared with naive mice, the percentage of TIGIT on CD44^hi^CD8^+^ T cells was still increased in memory mice (3.2 ± 0.5 vs. 5.1 ± 0.6, *P* = 0.03), although there was no difference in TIGIT expression on CD44^hi^CD4^+^ T cells between memory and naive mice (Supplemental Figure 1, J and K).

### TIGIT expression is higher on CD8^+^ T cells in memory versus previously naive mice during sepsis.

**Figure 1:**
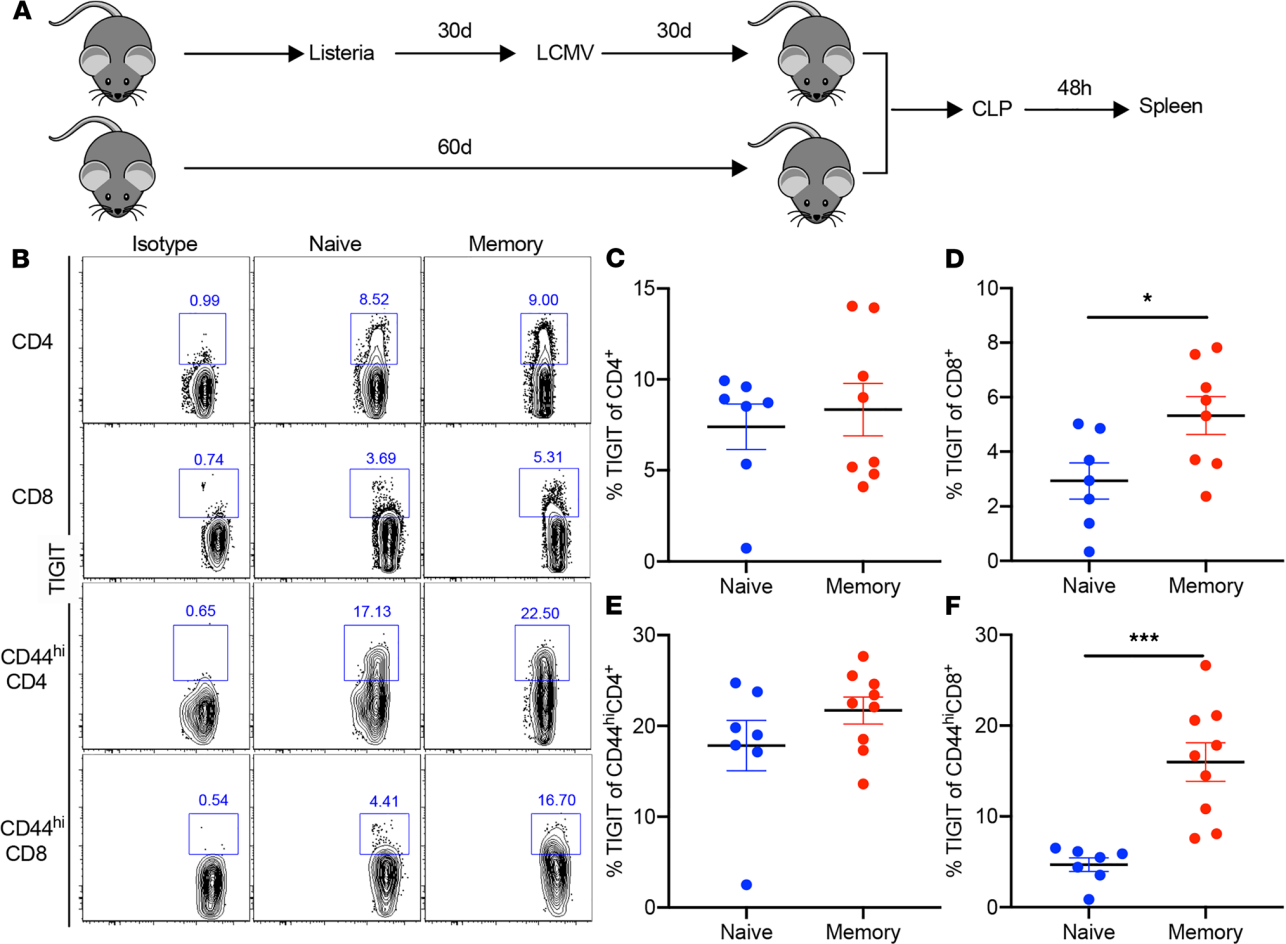
Coinhibitory molecule T cell Ig with ITIM domain expression is higher on CD8^+^ T cells in memory versus previously naive mice during sepsis. Naive B6 mice were infected with *Listeria monocytogenes* (LM) and with lymphocytic choriomeningitis virus (LCMV) i.p. 30 days later. Age-matched naive mice were used as controls. B6 naive and memory mice received cecal ligation and puncture (CLP) and were sacrificed at 48 hours after surgery. (**A**) Spleens were harvested and coinhibitory molecule T cell Ig with ITIM domain (TIGIT) expression on T cells was determined by flow cytometry. (**B**) Representative flow plots of TIGIT expression on bulk CD4^+^, CD8^+^, CD44^hi^CD4^+^, and CD44^hi^CD8^+^ T cells in previously naive and memory septic mice. (**C** and **D**) Summary data of the percentage of TIGIT on splenic CD4^+^ and CD8^+^ T cells in previously naive and memory mice (*n* = 7–8/group). (**E** and **F**) Summary data of the percentage of TIGIT on CD44^hi^CD4^+^ and CD44^hi^CD8^+^ T cells in spleens at 48 hours after CLP in previously naive versus memory mice (*n* = 7–8/group). Two groups were compared with the Mann-Whitney *U* test. *P < 0.05, ***P < 0.001. All data are shown as mean ± SEM and were pooled from 2 independent experiments.

To further determine TIGIT expression on T cells during sepsis, splenocytes from previously naive and memory mice that had undergone CLP surgery were analyzed by flow cytometry at 48 hours after CLP (Figure 1A). TIGIT expression on both bulk CD4^+^ and CD44^hi^CD4^+^ T cells in memory septic mice did not differ from that in previously naive septic mice (Figure 1, B, C, and E). In contrast, TIGIT expression was increased on bulk CD8^+^ T cells in memory mice compared with previously naive mice during sepsis (2.9% ± 0.6% vs. 5.3% ± 0.6%, *P* = 0.028, Figure 1, B and D). Further, TIGIT expression on CD44^hi^CD8^+^ T cells was significantly enhanced in memory mice relative to previously naive mice at 48 hours after CLP (4.6% ± 0.7% vs. 15.9% ± 2.1%, *P* = 0.0002, Figure 1, B and F).

### αTIGIT Ab worsens sepsis survival in memory but not previously naive hosts.

**Figure 2:**
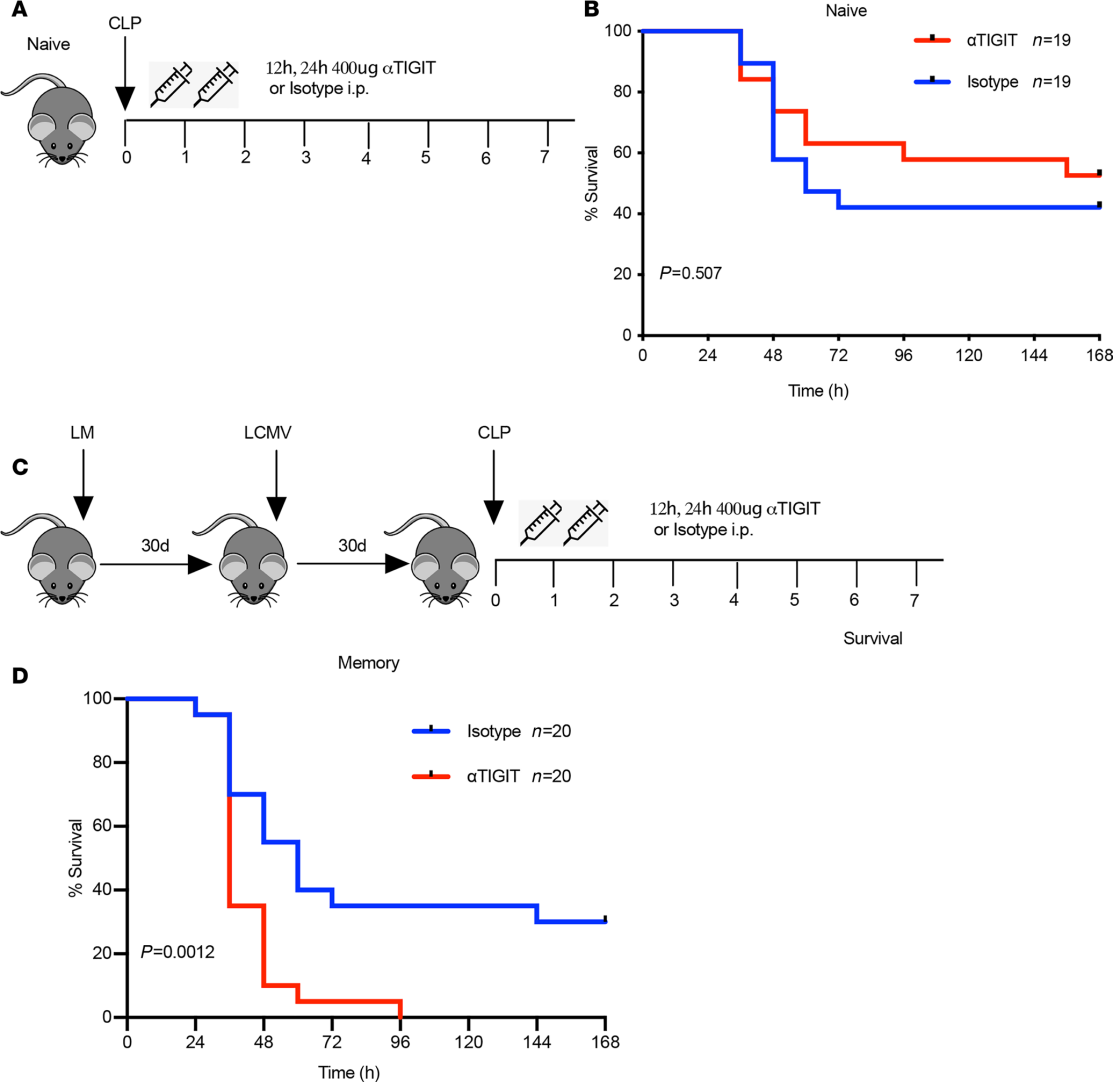
αTIGIT Ab significantly worsens sepsis survival in memory but not previously naive mice. (**A**) Schematic of experiment setup for CLP sepsis study in naive mice. (**B**) All naive mice received CLP surgery and were randomized to receive either αTIGIT Ab (*n* = 19) or isotype Ab (*n* = 19) at specified time points. All animals were monitored for 7-day survival. (**C**) Schematic of experiment setup for memory septic mice. (**D**) All memory mice received CLP surgery and were randomized to receive either αTIGIT Ab (*n* = 20) or isotype Ab (*n* = 20) at 12 and 24 hours after surgery. All animals were monitored for 7-day survival. Results represent 2 independent experiments. The log-rank (Mantel-Cox) test was used to test for significance.

First, we assessed the impact of blocking TIGIT signaling in previously naive mice by interrogating 7-day survival after CLP in the presence of treatment with the αTIGIT Ab or isotype control Ab (Figure 2A). We confirmed that this clone, previously reported to be blocking (18), was in fact blocking and not depleting at the concentrations used because in vivo administration of the Ab inhibited ex vivo staining with a fluorescently labeled anti-TIGIT, but without any change in CD4^+^ or CD8^+^ T cell counts in treated animals (Supplemental Figure 2). Results showed that αTIGIT Ab failed to significantly affect 7-day survival in previously naive septic mice (Figure 2B). Given the previous finding that TIGIT expressed on T cells was increased in memory septic mice compared with their previously naive counterparts, we hypothesized that treatment with αTIGIT Ab might confer a survival benefit in memory mice following sepsis. Memory mice underwent CLP and received either αTIGIT Ab or isotype control at 12 and 24 hours after CLP (Figure 2C). Strikingly, septic memory mice treated with αTIGIT exhibited dramatically decreased survival relative to isotype-treated mice (*P* = 0.0012, Figure 2D). Almost all memory mice treated with αTIGIT Ab died in the first 3 days following CLP.

### Apoptosis of memory T cells is increased by αTIGIT Ab in memory septic mice.

**Figure 3:**
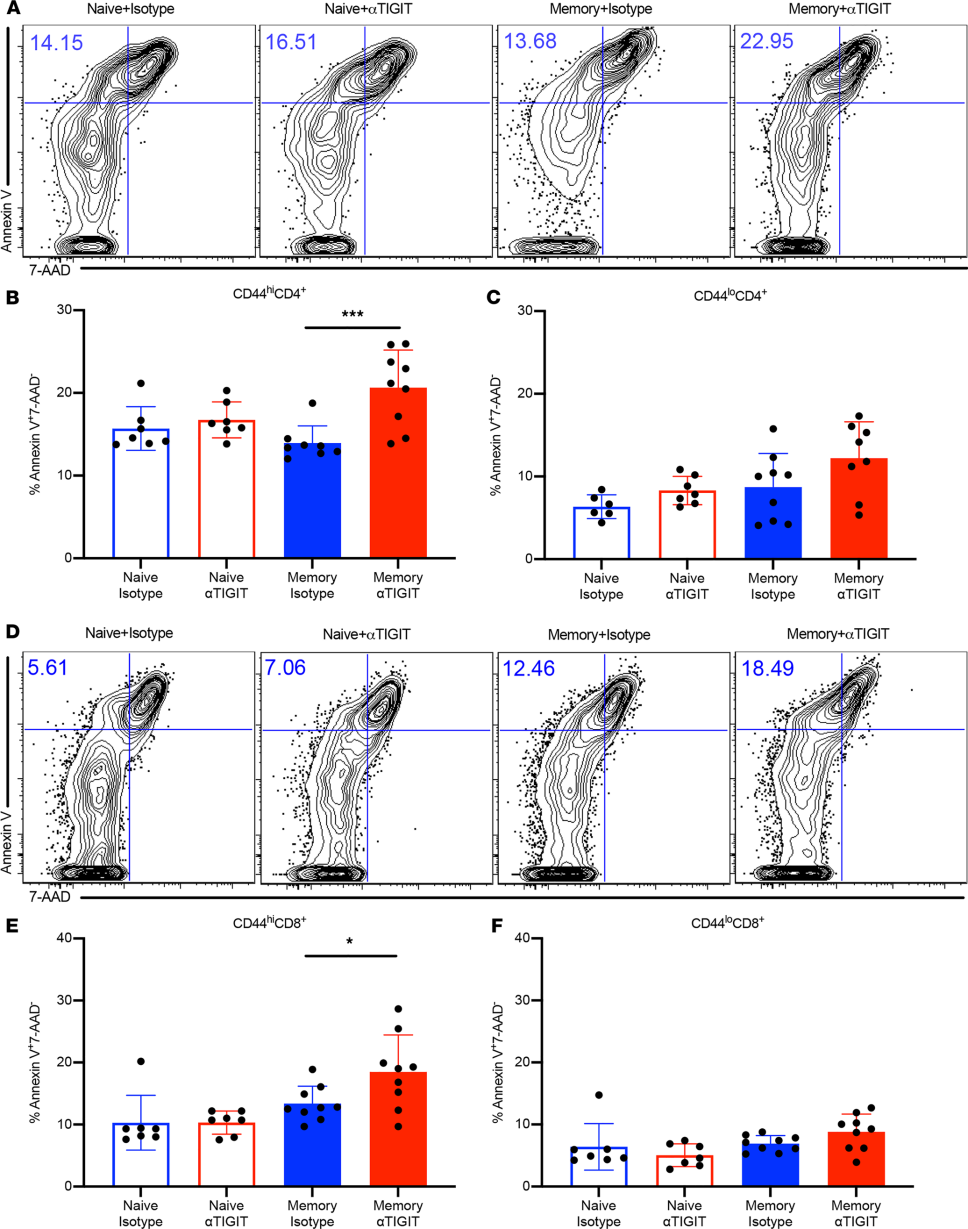
Apoptosis of CD44^hi^ memory T cells is accelerated by αTIGIT Ab in memory but not previously naive septic mice. Memory mice and age-matched naive controls received CLP, followed by injection of αTIGIT Ab or isotype control Ab at 12 and 24 hours after CLP. Mice were sacrificed and spleens were harvested at 48 hours after CLP. Splenocytes were stained with annexin V and 7-AAD for T cell apoptosis by flow cytometry. (**A**) Representative flow plots for annexin V^+^ and 7-AAD^–^ staining gated on CD44^hi^CD4^+^ T cells. (**B** and **C**) Summary data depicting frequency of apoptotic (annexin V^+^ 7-AAD^–^) CD44^hi^CD4^+^ and CD44^lo^CD4^+^ T cells in previously naive versus memory mice treated with αTIGIT Ab or isotype Ab (*n* = 7–9/group). (**D**) Representative flow plots for annexin V^+^ and 7-AAD^–^ staining gated on CD44^hi^CD8^+^ T cells. (**E** and **F**) Summary data of frequency of apoptotic CD44^hi^CD8^+^ and CD44^lo^CD8^+^ T cells among the 4 groups (*n* = 7–9/group). Groups were compared using 1-way ANOVA analysis and Tukey’s multiple comparison test. *P < 0.05, ***P < 0.001.

To further investigate the sharp early mortality in αTIGIT-treated memory septic mice, we first assessed apoptosis in the splenic T cell compartment in previously naive versus memory mice treated with αTIGIT Ab or isotype control at 48 hours following CLP (Figure 3A). Annexin V staining permits the detection of phosphatidylserine exposure on the cell membrane of apoptotic cells (19). Simultaneous staining of T cells with annexin V and 7-AAD allows the discrimination of early apoptotic (annexin V^+^ 7-AAD^–^) and late apoptotic or necrotic cells. Results showed that CD44^hi^CD4^+^ T cells in memory mice treated with αTIGIT Ab contained a higher frequency of early apoptotic T cells relative to isotype-treated memory controls during sepsis (20.6% ± 1.5% vs. 13.9% ± 0.7%, *P* = 0.0009, Figure 3B). In contrast, αTIGIT Ab had no effect on the apoptosis of CD44^lo^CD4^+^ T cells in either previously naive or memory mice with sepsis (Figure 3C). Within the CD8^+^ T cell compartment, αTIGIT resulted in increased frequencies of apoptotic cells among CD44^hi^CD8^+^ T cells relative to isotype control in memory but not previously naive septic mice (18.4% ± 1.9% vs. 13.4% ± 0.9%, *P* = 0.041, Figure 3, D and E). Conversely, there were no differences in the apoptosis of CD44^lo^ naive CD8^+^ T cells among the 4 groups (Figure 3F). Although memory septic animals possessed increased frequencies of late apoptotic (annexin V^+^ 7-AAD^+^) cells among both CD4^+^ and CD8^+^ CD44^hi^ memory T cells, frequencies of late apoptotic cells were not affected by anti-TIGIT in either the memory or previously naive septic mice (Supplemental Figure 3).

### αTIGIT Ab decreases frequencies of cytokine-producing T cells in memory septic mice.

**Figure 4:**
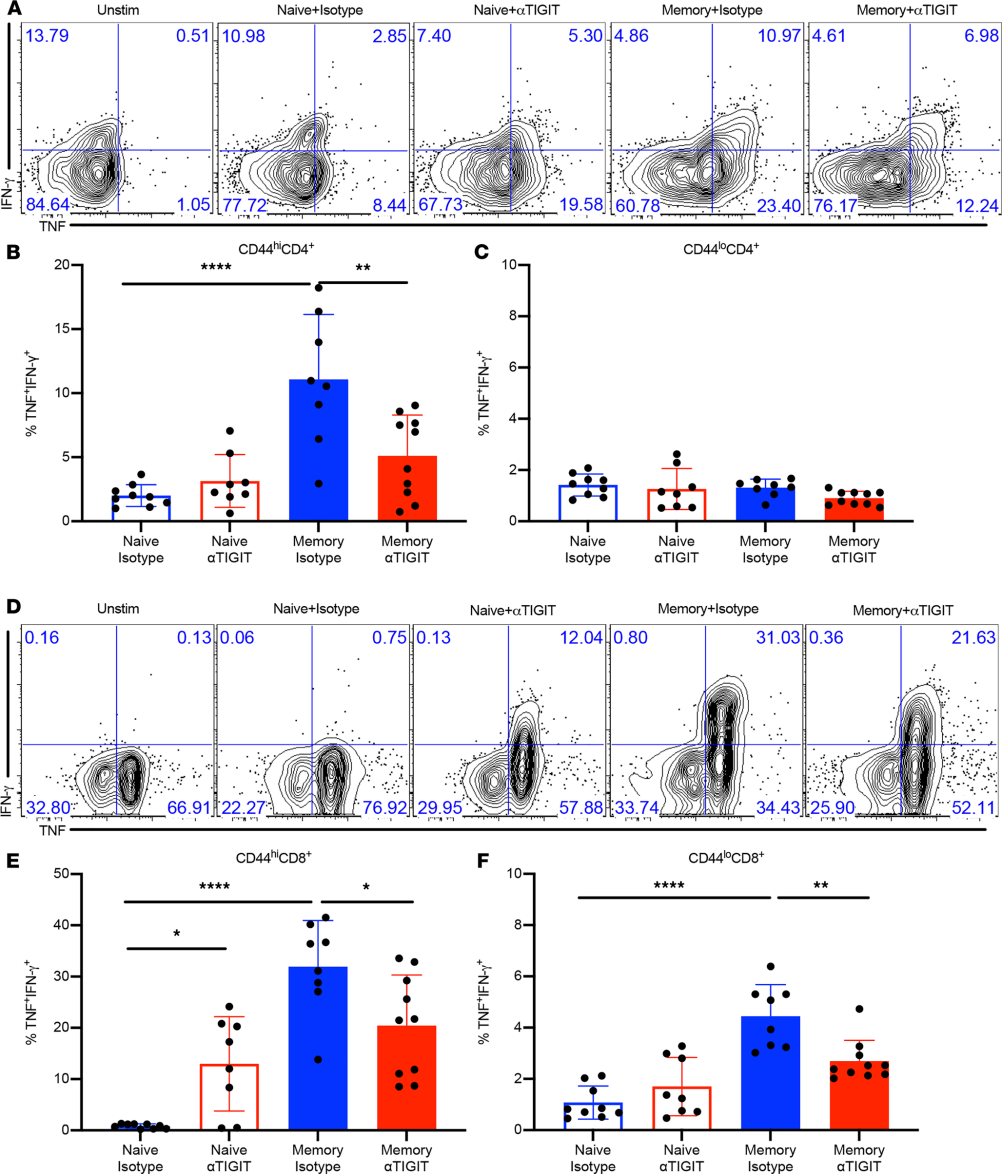
αTIGIT Ab decreases frequencies of cytokine-producing T cells in memory septic mice. Both naive and memory mice were subjected to CLP, received αTIGIT Ab or isotype control at 12 and 24 hours after CLP, and were sacrificed at 48 hours after CLP. Splenocytes were harvested and stimulated ex vivo with PMA and ionomycin for 4 hours, and then were assessed for TNF, IFN-γ production via intracellular cytokine staining. (**A**) Representative flow plots for TNF^+^ IFN-γ^+^ staining of CD44^hi^CD4^+^ T cells. (**B** and **C**) Summary figures for CD4^+^ T cell cytokine staining (*n* = 9–10/group). (**D**) Representative flow plots for TNF^+^ IFN-γ^+^ staining of CD44^hi^CD8^+^ T cells. (**E** and **F**) Summary figures for CD44^hi^CD4^+^ and CD44^hi^CD8^+^ T cell cytokine staining in 4 groups (*n* = 8–10/group). Results represent a minimum of 2 independent experiments. Groups were compared using 1-way ANOVA analysis and Tukey’s multiple comparison test. *P < 0.05, **P < 0.01, ****P < 0.0001.

To further investigate the effect of αTIGIT on T cell function, we assessed the frequencies of TNF- and IFN-γ secreting CD4^+^ and CD8^+^ T cells in memory versus previously naive mice treated with αTIGIT Ab or isotype control at 48 hours following CLP. Intracellular cytokine staining following ex vivo restimulation revealed that memory septic mice possessed increased frequencies of high-quality multipotent TNF^+^IFN-γ^+^–producing CD44^hi^CD4^+^ T cells compared with previously naive septic mice. Further, whereas αTIGIT Ab had no effect on the frequencies of TNF^+^IFN-γ^+^–producing cells among CD44^hi^CD4^+^ T cells in previously naive septic mice, it significantly decreased the frequencies of TNF^+^IFN-γ^+^–producing cells among CD44^hi^CD4^+^ T cells in memory septic mice (Figure 4, A and B). In contrast, αTIGIT Ab had no effect on the frequencies of TNF^+^IFN-γ^+^–producing cells within the CD44^lo^CD4^+^ T cell compartment in either previously naive or memory septic mice (Figure 4C). With regard to the CD8^+^ compartment, memory septic mice also possessed higher frequencies of TNF^+^IFN-γ^+^–producing CD44^hi^CD8^+^ T cells relative to previously naive septic mice. Interestingly, although αTIGIT Ab reduced the frequencies of TNF^+^IFN-γ^+^ CD44^hi^CD8^+^ T cells in memory septic mice, it paradoxically increased the frequencies of TNF^+^IFN-γ^+^–producing CD44^hi^CD8^+^ T cells in naive septic mice (Figure 4, D and E). Memory septic mice also possessed increased frequencies of TNF^+^IFN-γ^+^–producing cells among CD44^lo^CD8^+^ T cells compared with previously naive septic mice, and αTIGIT Ab decreased the frequencies of these CD44^lo^CD8^+^ TNF^+^IFN-γ^+^ cells in memory septic mice (Figure 4F). Taken together, these data indicate that αTIGIT Ab inhibited cytokine effector function of both CD4^+^ and CD8^+^ CD44^hi^ T cells in memory septic mice, but not in previously naive septic mice.

### αTIGIT results in decreased DNAM expression on CD44^hi^CD8^+^ T cells in memory but not previously naive septic mice.

**Figure 5:**
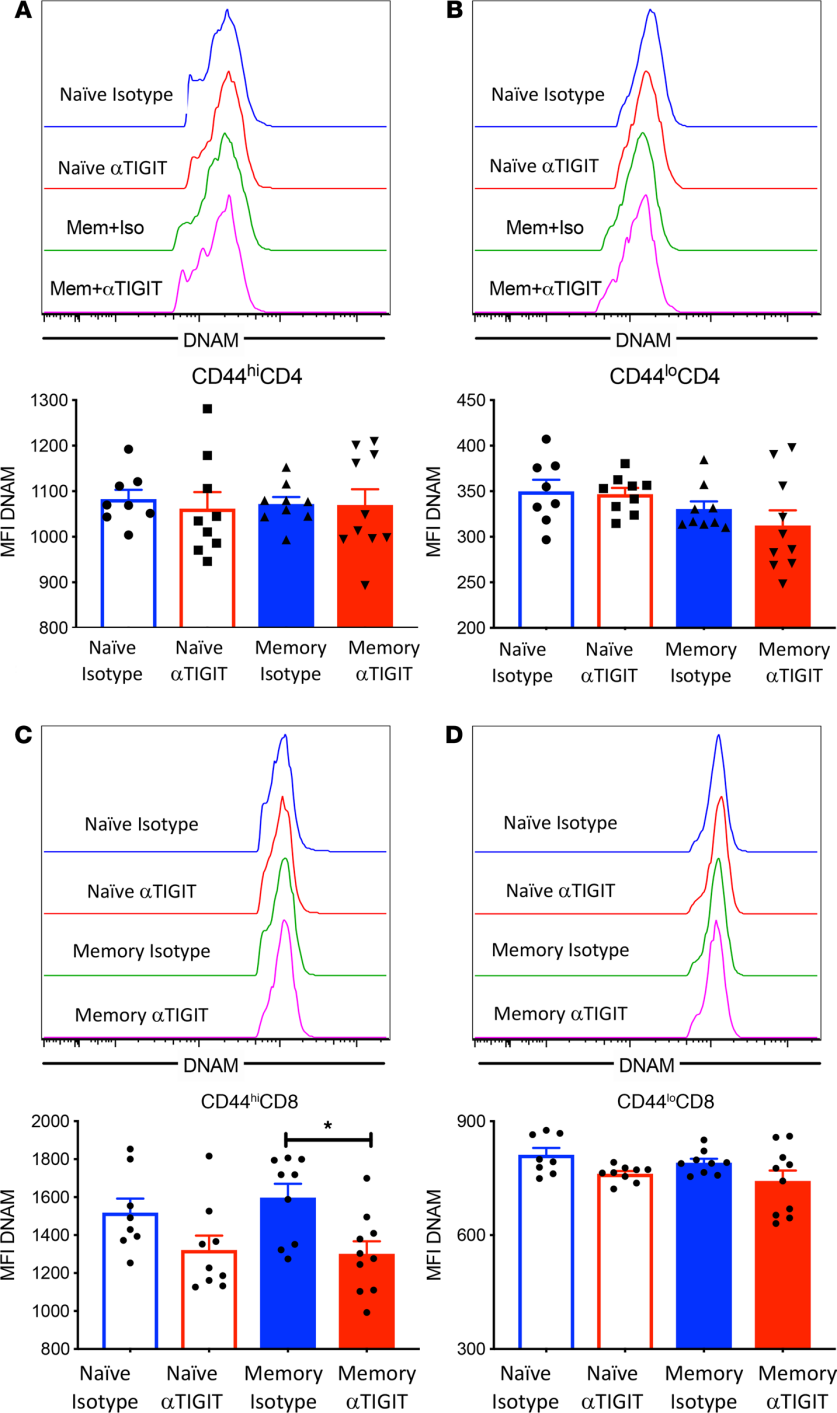
Memory CD8^+^ T cells exhibit decreased DNAM expression in αTIGIT-treated memory mice relative to memory controls. Memory and naive mice were subjected to CLP and received 2 doses of αTIGIT Ab or isotype control at 12 and 24 hours after CLP. Spleens were harvested at 48 hours after CLP and DNAM expression on T cells was assessed by flow cytometry. (**A** and **B**) Representative flow histograms and summary data depicting DNAM expression on CD44^hi^CD4^+^ and CD44^lo^CD4^+^ T cells. (**C** and **D**) Representative flow histograms and summary figure depicting DNAM MFI of CD44^hi^CD8^+^ and CD44^lo^CD8^+^ T cells, *n* = 8–10 in each group. Groups were compared using 1-way ANOVA analysis and Tukey’s multiple comparison test. *P < 0.05.

Next, we sought to explore the expression of DNAM, an activating receptor on T cells that competes with TIGIT for the same set of ligands, on T cells isolated from memory versus previously naive septic mice treated with either αTIGIT Ab or isotype control. No change in DNAM expression on either CD44^hi^CD4^+^ or CD44^lo^CD4^+^ T cells was observed among any of the 4 groups (Figure 5, A and B). In contrast, αTIGIT Ab resulted in decreased DNAM expression on CD44^hi^CD8^+^ T cells, but not CD44^lo^CD8^+^ T cells in memory septic mice (Figure 5, C and D). In contrast, there was no significant effect of αTIGIT Ab on DNAM expression on either CD44^hi^or CD44^lo^ CD8^+^ T cells in naive septic mice (Figure 5, C and D).

Given the synergistic nature of coinhibitory receptors, PD-1, 2B4, and TIM3 expression on memory and naive T cells were also assessed in memory and previously naive septic mice treated with αTIGIT or isotype control. PD-1 expression was increased on both CD44^hi^CD4^+^ and CD44^hi^CD8^+^ T cells in memory versus previously naive septic mice treated with αTIGIT Ab (Supplemental Figure 4, A, B, D, and E). No changes in PD-1 expression on CD44^lo^CD4^+^ or CD44^lo^CD8^+^ T cells were observed among the 4 groups (Supplemental Figure 4, C and F). Additionally, there were no significant differences in 2B4 or TIM-3 expression on memory or naive T cells among any of 4 groups (data not shown).

### Foxp3^+^ Tregs from αTIGIT-treated memory mice exhibit reduced activation and differentiation.

**Figure 6:**
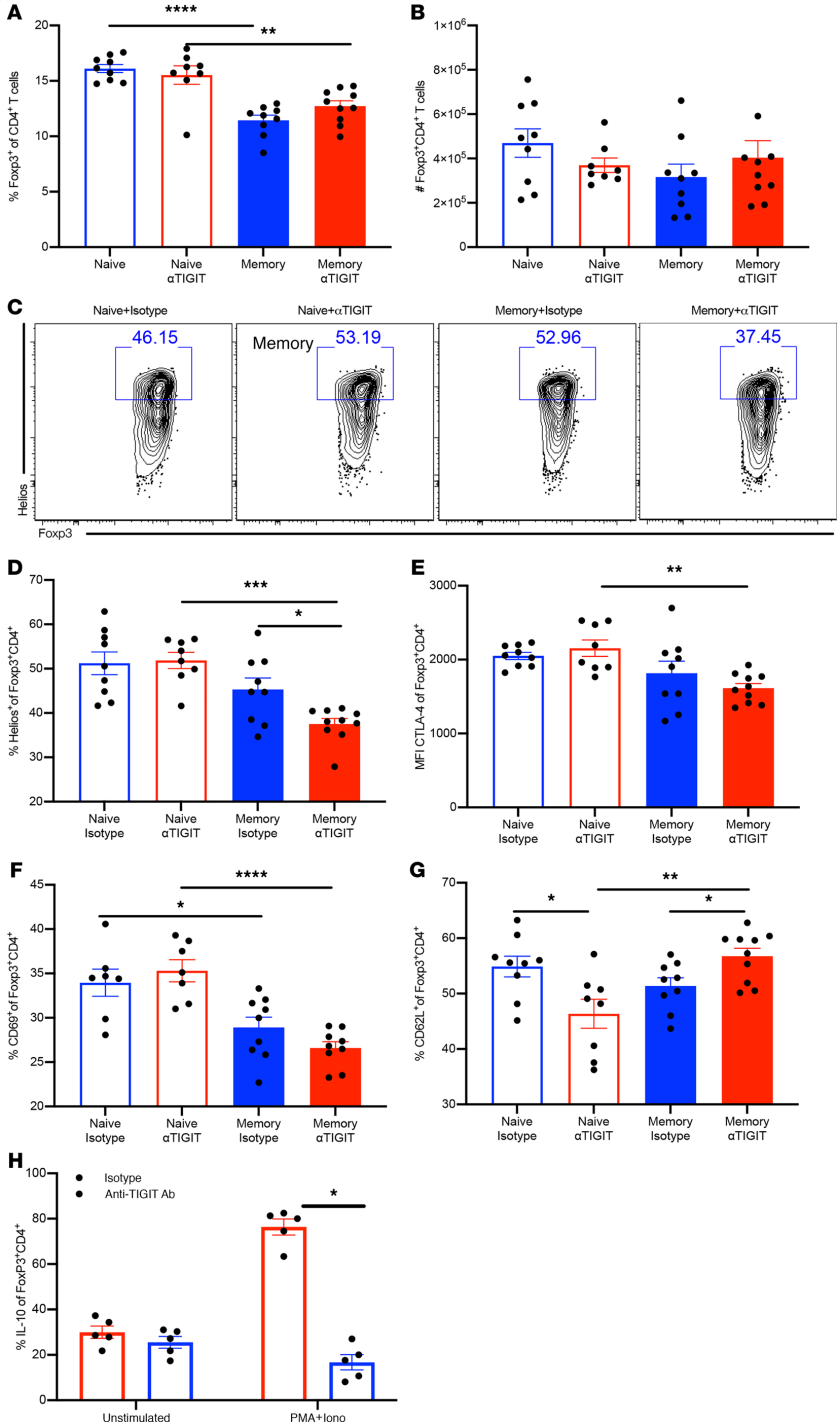
Foxp3^+^ Tregs from αTIGIT-treated memory mice exhibit deteriorated differentiation and activation. Splenocytes were harvested at 48 hours after CLP from memory and previously naive septic mice treated with αTIGIT or isotype Ab. (**A**) Summary data of the percentage of Foxp3^+^ Tregs in the 4 groups. (**B**) Absolute numbers of Foxp3^+^ Tregs among 4 groups. *n* = 8–9/group. Results were representative of 2 independent experiments. Groups were compared using 1-way ANOVA analysis and Tukey’s multiple comparison test. (**C**) Representative flow plots depicting Helios expression on CD4^+^Foxp3^+^ T cells. (**D**) Summary data of the percentage of Helios-expressing cells among Foxp3^+^ Tregs. (**E**) Summary figure depicting CTLA-4 MFI of Foxp3^+^ Tregs. (**F** and **G**) Summary data of the percentage of CD69^+^ and CD62L^+^ cells among Foxp3^+^ Tregs. (**H**) Splenocytes from memory septic mice were harvested at 48 hours and restimulated with PMA/ionomycin ex vivo for 4 hours and analyzed for IL-10. Results represent 2 independent experiments. Data are shown as the mean ± SEM. Groups were compared using 1-way ANOVA analysis and Tukey’s multiple comparison test. *P < 0.05, **P < 0.01, ***P < 0.001, ****P < 0.0001.

We next sought to characterize the effect of αTIGIT Ab on the differentiation and activation of Tregs in both memory and previously naive mice at 48 hours after CLP. Although there was no significant effect of αTIGIT Ab on the percentage and absolute numbers of Foxp3^+^ Tregs in either memory or previously naive groups (Figure 6, A and B), several aspects of Treg activation and differentiation were altered in anti-TIGIT–treated memory septic mice. First, the Ikaros family transcription factor Helios, which has been shown to be indicative of more stable and functional Tregs (20), was not affected in Tregs isolated from previously naive septic mice. In contrast, αTIGIT resulted in a significant decrease in the frequency of Helios of Foxp3^+^ Tregs in memory septic mice compared with isotype-treated memory controls (Figure 6, C and D). Cytotoxic T lymphocyte antigen 4 (CTLA-4) has been reported to be critically required for the function of Tregs in vivo (21). Our data demonstrated that Tregs isolated from αTIGIT-treated memory septic mice exhibit decreased CTLA-4 expression relative to Tregs isolated from αTIGIT-treated previously naive septic mice (Figure 6E).

The effect of αTIGIT on the activation of Tregs was also evaluated in both memory and naive mice. Memory septic mice possessed fewer frequencies of CD69^+^Foxp3^+^CD4^+^ Tregs relative to previously naive animals with sepsis, and treatment with αTIGIT had no effect on the CD69 expression of Tregs in memory septic mice at 48 hours after CLP (Figure 6F). Further, CD62L has been shown to be indicative of the resting status of Tregs (22). Strikingly, whereas αTIGIT significantly decreased CD62L expression on Tregs in previously naive mice with sepsis, it significantly increased CD62L expression on Tregs in memory mice with sepsis (Figure 6G). To address another major functional readout of Treg suppression, we assessed IL-10 secretion by Tregs that were isolated from memory septic mice treated with either isotype control or anti-TIGIT. Results indicated that Foxp3^+^ Tregs isolated from anti-TIGIT–treated memory septic mice contained significantly fewer IL-10 producers relative to Foxp3^+^ Tregs isolated from isotype-treated memory septic mice (Figure 6H). Taken together, these data indicate that αTIGIT results in less stable and less functional Tregs and inhibited the activation of Tregs in memory septic mice, while having no effect or the opposite effect on Tregs in previously naive septic mice.

### αTIGIT Ab differentially affected bacterial load and cytokines in peritoneal fluid in memory versus previously naive septic mice.

**Figure 7:**
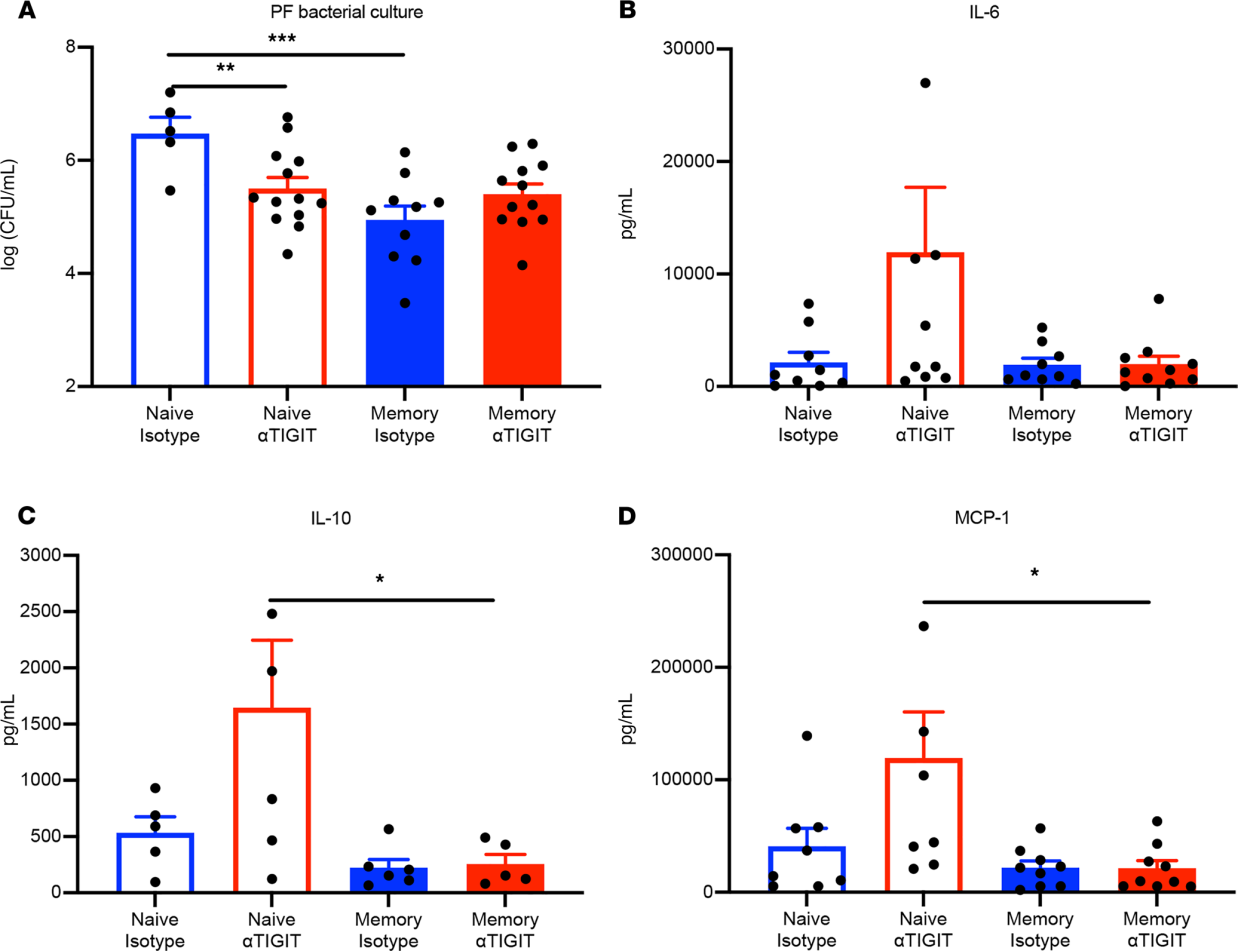
αTIGIT Ab differentially affected bacterial load and cytokines in peritoneal fluid in memory versus previously naive septic mice. Both previously naive and memory septic mice were administered αTIGIT Ab or isotype Ab at12 and 24 hours after CLP, and then were sacrificed at 36 hours after CLP and the sterile peritoneal fluid (PF) was taken for bacterial culture and cytokine detection. (**A**) Bacterial load in PF was measured in both memory and naive septic mice with or without αTIGIT. (**B–D**) Summary data of cytokines IL-6, IL-10, and MCP-1 as measured in the PF in the 4 groups. All data are representative of a minimum of 2 independent experiments. Groups were compared using 1-way ANOVA analysis and Tukey’s multiple comparison test. *P < 0.05, **P < 0.01, ***P < 0.001.

To determine the effect of αTIGIT Ab on bacterial clearance in the infectious site in memory versus previously naive mice with sepsis, we examined the bacterial load in peritoneal fluid (PF) at 36 hours after CLP. The 36-hour time point was chosen to capture relevant changes in systemic cytokines. Relative to previously naive septic mice, memory septic mice possessed significantly less bacterial load in the PF during sepsis. Further, whereas treatment with αTIGIT had no effect on bacterial load in memory septic mice, it significantly decreased bacterial load in the PF of previously naive mice at 36 hours after CLP (Figure 7A). Levels of circulating systemic inflammatory (IL1β, IL-6, TNF, IL-2, IL-13, and MCP-1) as well as antiinflammatory (IL-10) cytokines were also evaluated 36 hours after CLP in memory versus previously naive mice. Compared with isotype-treated previously naive controls, IL-6, IL-10, and MCP-1 serum levels exhibited a trend toward increase in αTIGIT-treated previously naive mice (IL-6: *P* = 0.13; IL-10: *P* = 0.13; and MCP-1: *P* = 0.07). Although serum IL-10, IL-6, and MCP-1 were lower in the anti-TIGIT–treated memory mice relative to anti-TIGIT–treated previously naive mice (Figure 7, B–D), results indicated no significant differences in the anti-TIGIT–treated versus isotype control-treated memory septic animals in any of the cytokines tested (Figure 7, B–D, and data not shown). Moreover, analysis of an additional time point at 48 hours after CLP revealed no significant differences between the anti-TIGIT–treated versus control groups in either the previously naive or memory septic animals in any of the cytokines tested (Supplemental Figure 5). Further, there were no statistically significant differences in the absolute numbers of CD4^+^ or CD8^+^ T cell populations (Supplemental Figure 6A), or in any of the innate immune subsets (CD11c^+^ DCs, CD11b^+^ myeloid cells, F4/80^+^ macrophages, NK1.1^+^ NK cells, or Gr-1^+^ neutrophils, Supplemental Figure 6B) within the PF of anti-TIGIT– versus isotype-treated memory mice. There were also no differences in the absolute numbers of CD11c^+^ DCs, CD11b^+^ monocytes, F4/80^+^ macrophages, and NK1.1^+^ NK cells in splenocytes of anti-TIGIT–treated versus control septic memory mice (Supplemental Figure 6C).

## Discussion

In the present study, we demonstrate fundamental differences in the role of TIGIT coinhibitory signaling during sepsis in previously naive versus immunologically experienced murine hosts. Consistent with a previous report, our data demonstrated that TIGIT was upregulated on memory T cells both before and during sepsis (6). Naive mice possessed fewer memory T cells and exhibited less TIGIT expression on CD4^+^ and CD8^+^ T cells (Supplemental Figure 1, H and I). We submit that this immunologically experienced mouse model, generated via sequential infection of mice with acutely cleared bacterial and viral pathogens, better recapitulates relevant aspects of the human immune system. Thus, the fact that αTIGIT Ab had no impact on the survival in previously naive septic mice, whereas it sharply worsened survival in memory mice with sepsis, has important implications for the role of TIGIT during human immune responses in sepsis. Mechanistically, αTIGIT increased apoptosis and decreased cytokine effector function of CD44^hi^ T cells in memory but not previously naive mice. Additionally, αTIGIT diminished the stability and activity of Tregs during sepsis in memory mice, but not in previously naive septic mice.

TIGIT has emerged as a crucial coinhibitory receptor in studies of antitumor and antiviral immune responses (23, 24). An initial study demonstrated that TIGIT exerted immunosuppressive effects by triggering CD155 in DCs, thereby preventing DC maturation and inducing IL-10 production (12). Other studies revealed that TIGIT can directly inhibit T cell proliferation and cytokine production independent of antigen-presenting cells (APCs) (25). Immune suppression characterized by increased expression of coinhibitory receptors plays a crucial role in the immunopathological changes of sepsis, which results in secondary infection and worse outcome (26). Recently, checkpoint blockade therapies have garnered attention in the area of clinical research. Anti-TIGIT alone or in combination with PD-1 blockade has emerged as a promising therapy in antitumor treatment (13, 27). We also demonstrated that anti-TIGIT Ab (clone 1G9) improved survival in septic mice with preexisting malignancy (unpublished observations), a situation characterized by immune activation and development of effector/memory T cells (28). In the study, results showed that the blockade of TIGIT pathway in naive septic mice improved T cell function by increasing the frequencies of TNF^+^IFN-γ^+^–producing T cells. This increased T cell function was associated with improved control of bacterial burden in the PF. Notably, this improved bacterial control was not associated with a survival benefit in previously naive septic mice, suggesting that mortality in this model is mediated by immune dysregulation and not from bacterial overgrowth per se, a concept that has been repeatedly supported in studies of sepsis in mice and humans. What then underlies the observed disparate effects of αTIGIT Ab on sepsis survival in memory versus naive mice? Joller and colleagues demonstrated that TIGIT engagement can limit T cell–driven inflammation and protect against immune pathology via induction of immune-modulatory cytokine IL-10 in acute viral infection model (29). Because memory mice possess more activated memory T cells with higher frequencies of proinflammatory cytokine producers and are better able to control bacterial load relative to naive mice during sepsis, we posit that the elevated TIGIT expression on memory T cells would normally function to limit the overactivation of T cells and immunopathological injury in memory septic mice. Blockade of TIGIT signaling may therefore lead to overactivation of memory T cells, resulting in activated-induced apoptosis and dysfunction of T cells, and contributing to worsened survival in memory septic mice. It is important to note that although a recent study in a cancer model attributed the function of anti-TIGIT to its action on NK cells (30), we found no differences in the number of NK cells between the isotype versus anti-TIGIT–treated groups for either previously naive or memory septic animals (data not shown).

Although TIGIT is expressed at higher frequency among CD4^+^ T cells, the difference in frequency of TIGIT^+^ cells between naive and memory mice is more pronounced among CD8^+^ T cell populations. These data suggest that TIGIT^+^ CD8^+^ T cells may represent a specific differentiation program, and that TIGIT could be playing a cell autonomous role on these cells. For example, our data also demonstrated that αTIGIT resulted in decreased DNAM expression on CD8^+^ T cells, which itself could lead to increase mortality in memory mice with sepsis. This possibility is consistent with a recent study that showed CD226^hi^CD8^+^ T cells are required for the efficacy of anti-TIGIT immunotherapy in a tumor model (31). In our study, although αTIGIT affected the expression of this costimulatory receptor, it had no effect on the expression of numerous coinhibitory molecules, including PD-1, 2B4, and TIM-3 on CD44^hi^ or CD44^lo^ T cells either in previously naive or memory mice with sepsis. These findings are in line with studies showing that anti-TIGIT did not significantly impact PD-1 expression by antigen-specific CD8^+^ T cells in patients with melanoma (32). However, they are in opposition to another report showing that Ab-mediated TIGIT blockade resulted in significant downregulation of PD-1 and TIM-3 expression during chronic viral infection (29). Thus, further investigation into the conditions under which TIGIT blockade affects other cosignaling molecule expression and signaling are required.

In addition to its expression and cell-autonomous effect on effector T cells, TIGIT is also expressed on Tregs, where it serves to promote Treg suppressor function during sepsis (27). Recent work shows that TIGIT expression marks a subset of Tregs that exhibit higher Treg effector molecules (i.e., CTLA-4) and enhanced suppressive capacity in vitro (33, 34). Further, Helios expression distinguishes thymus-derived Tregs from peripherally induced ones and is also indicative of more stable and functional Tregs (35). The fact that αTIGIT-induced reduction in Helios expression in Tregs in memory septic mice is associated with increased mortality in sepsis is line with published reports showing that Tregs are beneficial during the early, high-inflammatory stage of sepsis (36, 37). Thus, in addition to cell-autonomous effects on memory T cells, anti-TIGIT could be indirectly affecting T cell apoptosis and effector function through its effect on Treg function, possibly warranting further investigation.

Our study is limited by the fact that we used a model of CLP, a mouse model that is widely thought to approximate the situation of ruptured appendicitis in humans. Because sepsis as it is defined clinically can arise from a number of different infectious etiologies, including respiratory and urinary tract infections, the findings in this model of peritonitis may not be representative of other sepsis etiologies. Indeed, additional work in other models of sepsis, and in human septic patients, will be required to fully elucidate the role of TIGIT in sepsis-induced immune dysregulation.

The differential roles that TIGIT plays in previously naive versus immunologically experienced hosts is an essential consideration for the development of immunotherapeutic approaches involving the TIGIT blockade. Because our data showed that blockade of TIGIT pathway triggered the apoptosis and dysfunction of effector T cells, as well as dampened the function of Tregs in immunologically experienced host during sepsis, we conclude that rather than promoting T cell exhaustion and dysfunction like other coinhibitory receptors, TIGIT might play a protective role in preventing T cell apoptosis and preserving T cell function during sepsis. Finally, this study sheds light on the complexity and contradiction of targeting coinhibitory receptor during sepsis. Therefore, further investigation is required to determine whether the enhancement of the TIGIT pathway could confer a survival benefit in immunologically experienced hosts during sepsis, and how TIGIT coinhibitory signaling interacts with PD-1, 2B4, and TIM-3 on both effector and Tregs during sepsis.

## Methods

### Memory mice model.

Six-week-old male and female C57BL/6J (B6) mice were purchased from The Jackson Laboratory. All animals were housed in the BSL-2 facility of Emory University and maintained following Emory IACUC guidelines (protocol 2003238-082518). For the memory mouse model, 1 × 10^4^ CFU of *L*. *monocytogenes* were i.p. injected. After 30 days, these mice were i.p. injected with a single dose of 2 × 10^5^ PFU of the Armstrong strain of LCMV. Titers of the virus were determined by plaque assay on Vero cells. The model was established after another 30 days after LCMV. The memory mice generated via this protocol were bled and assessed0, 10, 25, 40, and 56 days after *L*. *monocytogenes* infection by flow cytometry. Both of these acute infections were cleared on day 59 after *L*. *monocytogenes* infection. Age- and sex-matched B6 laboratory mice, termed naive mice, served as controls and were maintained in the same BSL-2 facility for 60 days before surgery.

### CLP.

CLP was performed 60 days after *L*. *monocytogenes* infection. Under isoflurane anesthesia, a midline incision was performed, and the cecum was externalized. The cecum was ligated with a 4-0 silk suture and punctured through and through with a 25-gauge needle. All animals received buprenorphine (0.1 mg/kg; McKesson Medical) preoperatively for pain relief. Immediately after surgery, animals received 1 mL sterile saline for fluid resuscitation as well as antibiotics (50 mg/kg ceftriaxone and 35 mg/kg metronidazole, Sigma-Aldrich) every 12 hours for 2 days. Mice were randomized to receive 400 μg anti-TIGIT blocking mAb (clone 1G9, BioXcell) (18) or isotype control Ab (mouse IgG, clone MOPC-21, BioXcell) via i.p. injection at 12 and 24 hours after CLP. All animals were euthanized with CO_2_ asphyxiation at the indicated time points.

### Flow cytometry.

Spleens were harvested after mice were sacrificed 48 hours after CLP, and then were processed to single-cell suspensions though a 70 μm filter. Splenocytes were rinsed with 10 mL cold PBS, and 200 μL from each spleen was put into a 96-well plate for staining. Anti-CD3 (BD, clone 500A2), anti-CD4 (clone RM4-5, BioLegend), anti-CD8 (clone MCD0830, Invitrogen), anti-CD44 ( clone IM7, BioLegend), anti-CD226 (clone 10E5, BioLegend), anti-PD-1 (clone 29F.1A12, BioLegend), anti-2B4 (clone eBio244F4, Thermo Fisher Scientific), and anti-TIM-3 (clone RMT3-23, BioLegend) were used for surface staining to determine T cell phenotype. For the detection of cell apoptosis, splenocytes were stained with a FITC Annexin V apoptosis detection kit with 7-AAD (BioLegend). Tregs were identified via intracellular staining for Foxp3-APC (clone FJK-16S, eBioscience). Splenocytes were surface stained for anti-CD62L (MEL-14), anti-CD69 (H1.2F3), and then permeabilized using a Foxp3 kit (BD Biosciences) and stained with anti-Foxp3, anti-CTLA-4 (UC10-489), and anti-Helios (22F6, all Abs from BioLegend). AccuCheck Counting Beads (Thermo Fisher Scientific) were added after staining to calculate the absolute number of cells per spleen.

### Intracellular cytokine staining.

For intracellular cytokine staining, splenocytes were stimulated with 30 ng/mL PMA and 1 μg/mL ionomycin in the presence of GolgiPlug (BD Pharmingen) for 4 hours at 37°C, and then permeabilized and fixed using BD Cytofix/Cytoperm kit followed by staining with anti-TNF (clone MP6-XT22, BioLegend) and anti-IFN-γ (clone XMG1.2, BioLegend). Samples were analyzed on an LSRII flow cytometer (BD), and data were analyzed using FlowJo software (version 9.9.6).

### Bacterial culture and cytokines in peritoneal fluid.

Peritoneal fluid (PF) samples were obtained by abdominal lavage with 3 mL sterile saline at 36 hours after CLP. A total of 100 μL PF was taken for detecting bacterial load, which were serially diluted in sterile saline and cultured on sheep’s blood agar plates (Remel). Plates were incubated overnight at 37°C in 5% CO_2_, and colony counts were determined on plates receiving 1:10^4^ diluted inoculum. The rest of the PF samples were centrifuged at 4°C with 120 *g* for 10 minutes. Cytokine concentration was determined using the Bio-Plex 200 System according to the manufacturer’s instructions (Bio-Rad). IL-6, IL-10, MCP-1 levels were reported in pg/mL. All samples were run in duplicate. Results were analyzed using Bio-Plex Manager 3.0 software.

### Statistics.

Data are shown as the mean ± SEM. One-way ANOVA and multiple comparison tests were used to compare the differences among the 4 groups. Mann-Whitney *U* tests were used to compare continuous variables between 2 groups. All statistical analyses were conducted using GraphPad Prism 8.0 software. Two-tailed *P* values of less than 0.05 were considered significant.

### Study approval.

This study was conducted under approval from the Emory University IACUC (protocol 2003238-082518).

## Author contributions

MLF and CMC designed the research studies. YS, TY, HS, MX, DL, and ZL conducted the experiments and the acquired data. YS and HS analyzed the data. MLF and YS wrote the manuscript. All authors edited the manuscript.

## Supplementary Material

Supplemental data

## Version 1 03/08/2021

Electronic publication
