# Supplementary material for: Anti-TIGIT differentially affects sepsis survival in immunologically experienced versus previously naive hosts
Source: JCI Insight. 2021 Mar 8;6(5):e141245. doi: 10.1172/jci.insight.141245 (PMC8021109; doi:10.1172/jci.insight.141245)
Supplement: Supplemental data [file jciinsight-6-141245-s190.pdf]

Supplementary Material

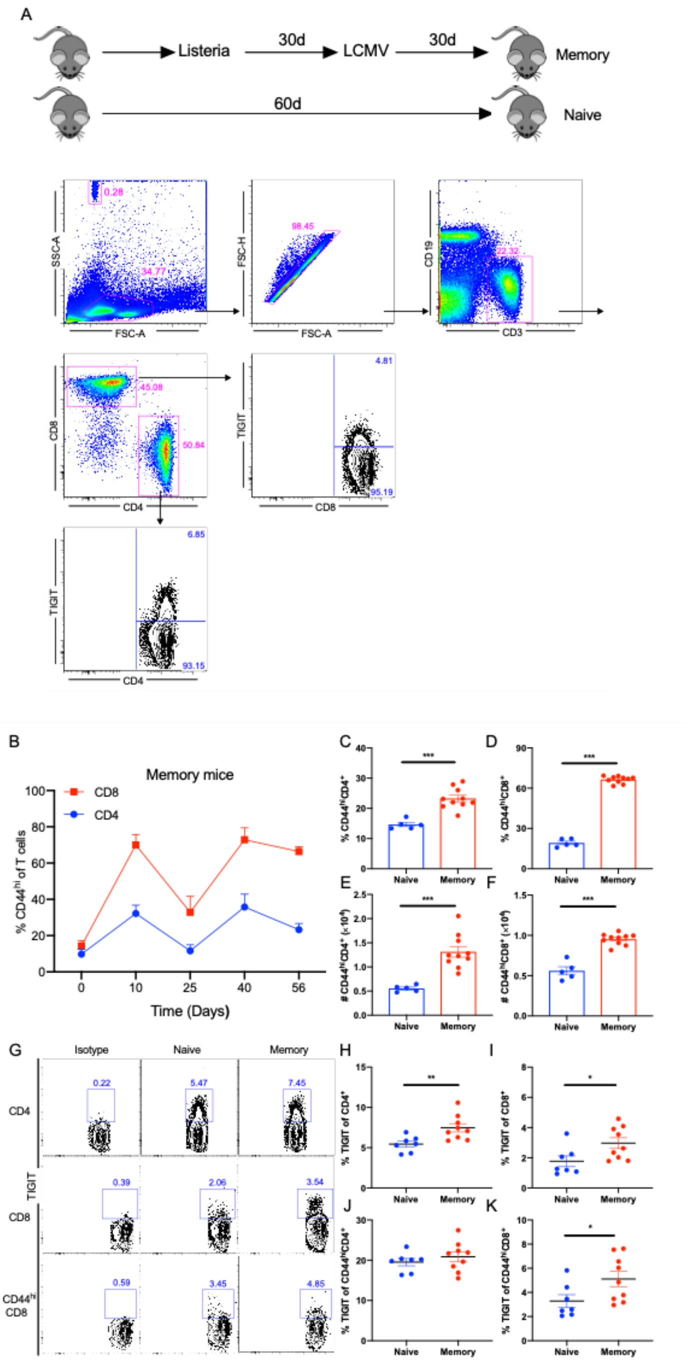

Supplemental Figure 1. TIGIT expression on both $CD4^+$ and $CD8^+$ T cells is upregulated in memory mice relative to naïve mice. Naïve B6 mice were infected with *Listeria monocytogenes* (LM) and were infected with LCMV intraperitoneally 30 days later. Age-matched naïve mice were used as controls (A). Representative flow plots of the gating strategy to identify the TIGIT expression on $CD4^+$ and $CD8^+$ T cells in the spleen of memory mice. The frequency of memory ($CD44^{hi}$) T cells was assessed on d0, d10, d25, d40, d56 post-LM by flow cytometry. (B) Expansion of $CD44^{hi}$ $CD4^+$ and $CD44^{hi}$ $CD8^+$ T cells in the blood over time following antigen exposure (n=10/group). (C-D) Summary of frequency of $CD44^{hi}CD4^+$ T cells and $CD44^{hi}$ $CD8^+$ T cells in naïve mice compared with memory mice on d56 following LM infection (n=5-10/group). (E-F) Absolute numbers of $CD44^{hi}$ $CD4^+$ T cells and $CD44^{hi}CD8^+$ T cells in the blood on d56 following LM infection (n=5-10/group). (G) Representative flow plots of TIGIT expression on $CD4^+$, $CD8^+$, and $CD44^{hi}$ $CD8^+$ T cells. (H-I) Summary data of the percentage of TIGIT on bulk $CD4^+$ and $CD8^+$ T cells in spleen in naïve and memory mice (n=7-9/group). (J-K) Summary data of the percentage of TIGIT on $CD44^{hi}$ $CD4^+$ and $CD44^{hi}$ $CD8^+$ T cells in the spleens in memory versus naïve mice (n=7-9/group). Two groups were compared with the Mann-Whitney nonparametric test. *, p<0.05. **, *p*<0.01. ***, *p*<0.001. All data expressed as mean ± SEM and were pooled from two independent experiments.

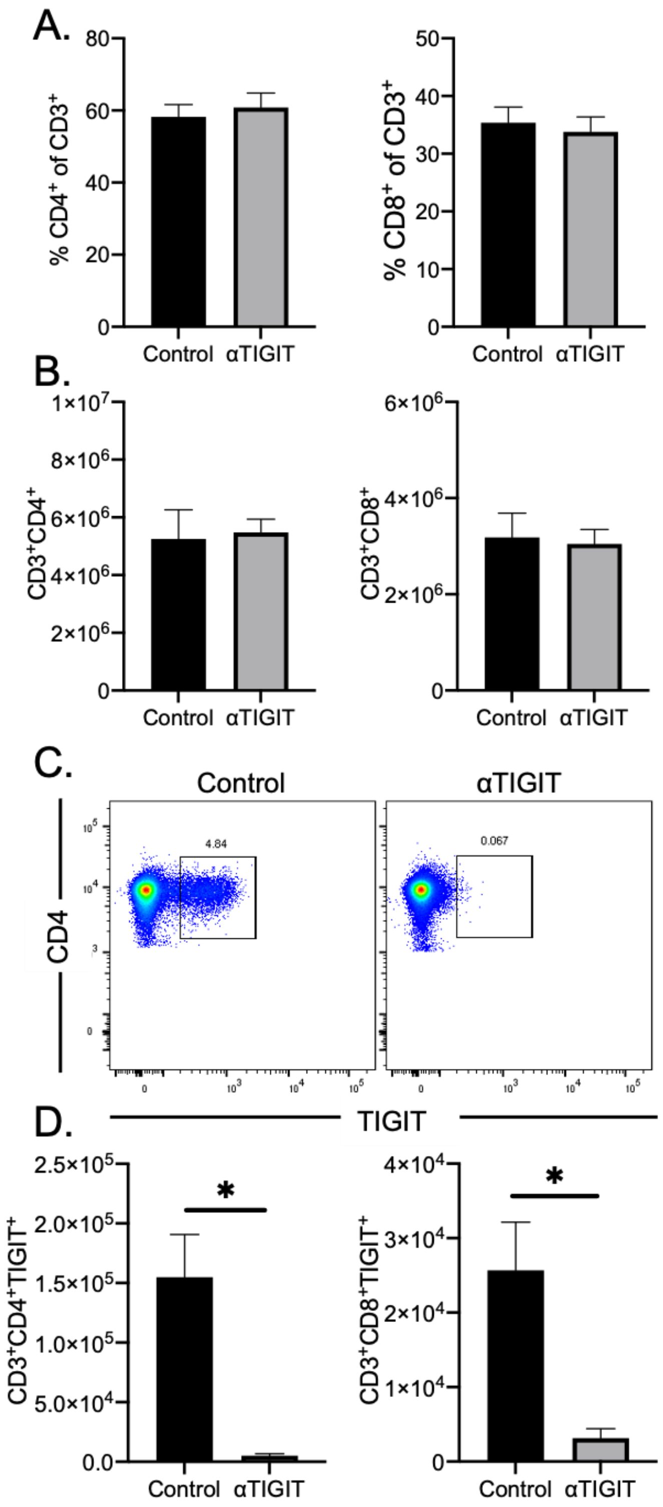

Supplemental Figure 2. Anti-TIGIT Clone 1G9 is a blocking antibody. Memory septic mice were injected with anti-TIGIT in Figure 2 and splenocytes were stained with anti-TIGIT. Frequencies and absolute numbers of $CD4^+$ and $CD8^+$ T cells were analyzed. Two groups were compared with the Mann-Whitney nonparametric test. *, $p<0.05$. All data expressed as mean ± SEM and were pooled from two independent experiments.

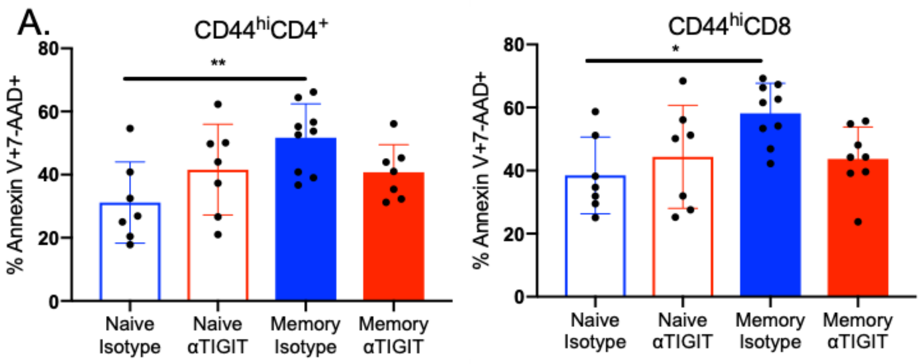

Supplemental Figure 3. Frequencies of AnnexinV$^+$7AAD$^+$ late apoptotic cells are not affected in previously naïve or memory septic mice following anti-TIGIT antibody administration. Memory mice and age-matched naïve controls received CLP, followed by injection of αTIGIT Ab or isotype control Ab at 12h and 24h post-CLP. Mice were sacrificed and spleens were harvested at 48h after CLP. Splenocytes were stained with Annexin V and 7-AAD for T cell apoptosis by flow cytometry. (A) Summary data depicting frequency of late apoptotic (AnnexinV$^+$7-AAD$^+$) CD44^{hi}CD4$^+$ and (B) CD44^{hi}CD8$^+$ T cells in previously naïve vs. memory mice treated with αTIGIT Ab or isotype Ab (n=7-9/group). Groups were compared using one-way ANOVA analysis and Turkey multiple comparison test. *, p<0.05, **, p<0.01.

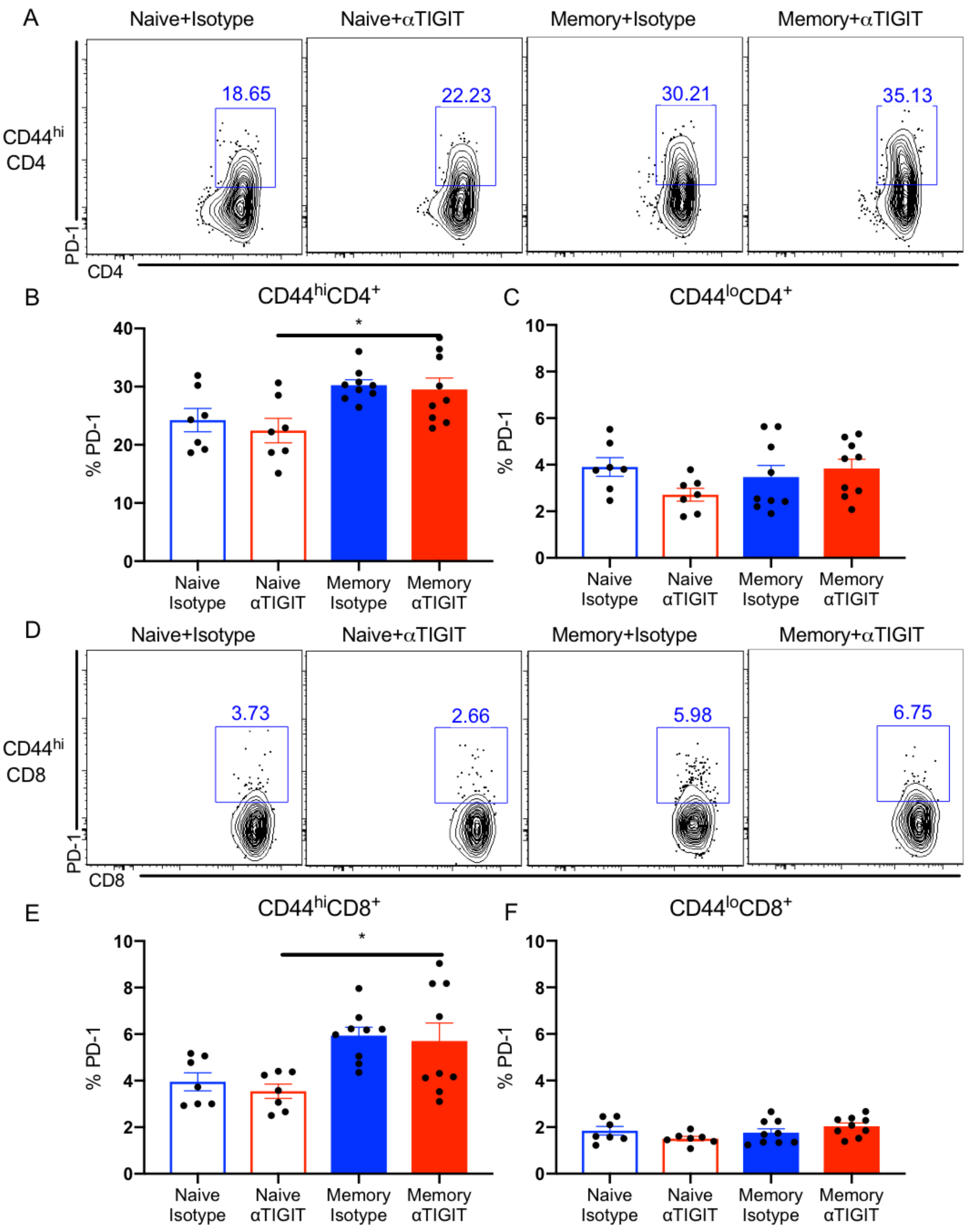

Supplemental Figure 4. Memory T cells from memory mice treated with αTIGIT Ab exhibit the increase of PD-1 compared with αTIGIT-treated previously naïve mice. Splenic T cells were harvested and the expression of PD-1 was assessed at 48h after CLP. (A) Representative flow plots for PD-1 on CD44^{hi}CD4^{+} T cells. (B-C) Summary data of the percentage of PD-1 on CD44^{hi}CD4^{+} and CD44^{lo}CD4^{+} T cells. (D) Representative flow plots for PD-1 on CD44^{hi}CD8^{+} T cells (n=7-9/group). (B-C) Summary data of the percentage of PD-1 on CD44^{hi}CD8^{+} and CD44^{lo}CD8^{+} T cells (n=7-9/group). Results were representative of two independent experiments. Error bars represent mean ± SEM. Groups were compared using one-way ANOVA analysis and Turkey multiple comparison test. *, $p<0.05$.

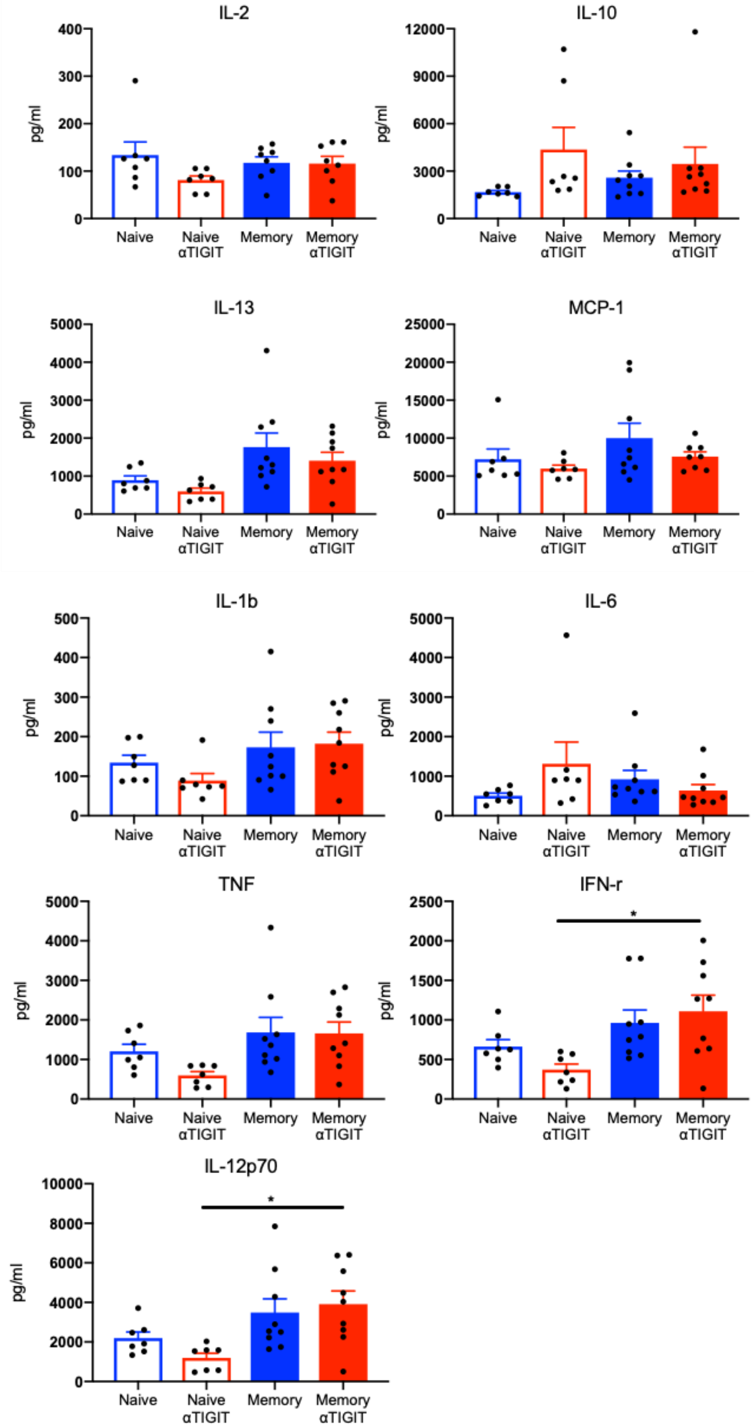

Supplemental Figure 5. αTIGIT Ab does not affect cytokines in peritoneal fluid at 48h post CLP in memory vs. previously naïve septic mice. Both previously naïve and memory septic mice were administered αTIGIT Ab or isotype Ab at 12h and 24h post-CLP, and then were sacrificed at 48h after CLP and the sterile peritoneal fluid was obtained for cytokine detection. Summary data of cytokines IL-2, IL-10, IL-13, MCP-1, IL-1beta, IL-6, TNF, IFN-γ, and IL-12p70 are shown as measured in the peritoneal fluid in the four groups. All data depicted a minimum of two independent experiments. Groups were compared using one-way ANOVA analysis and Turkey multiple comparison test.

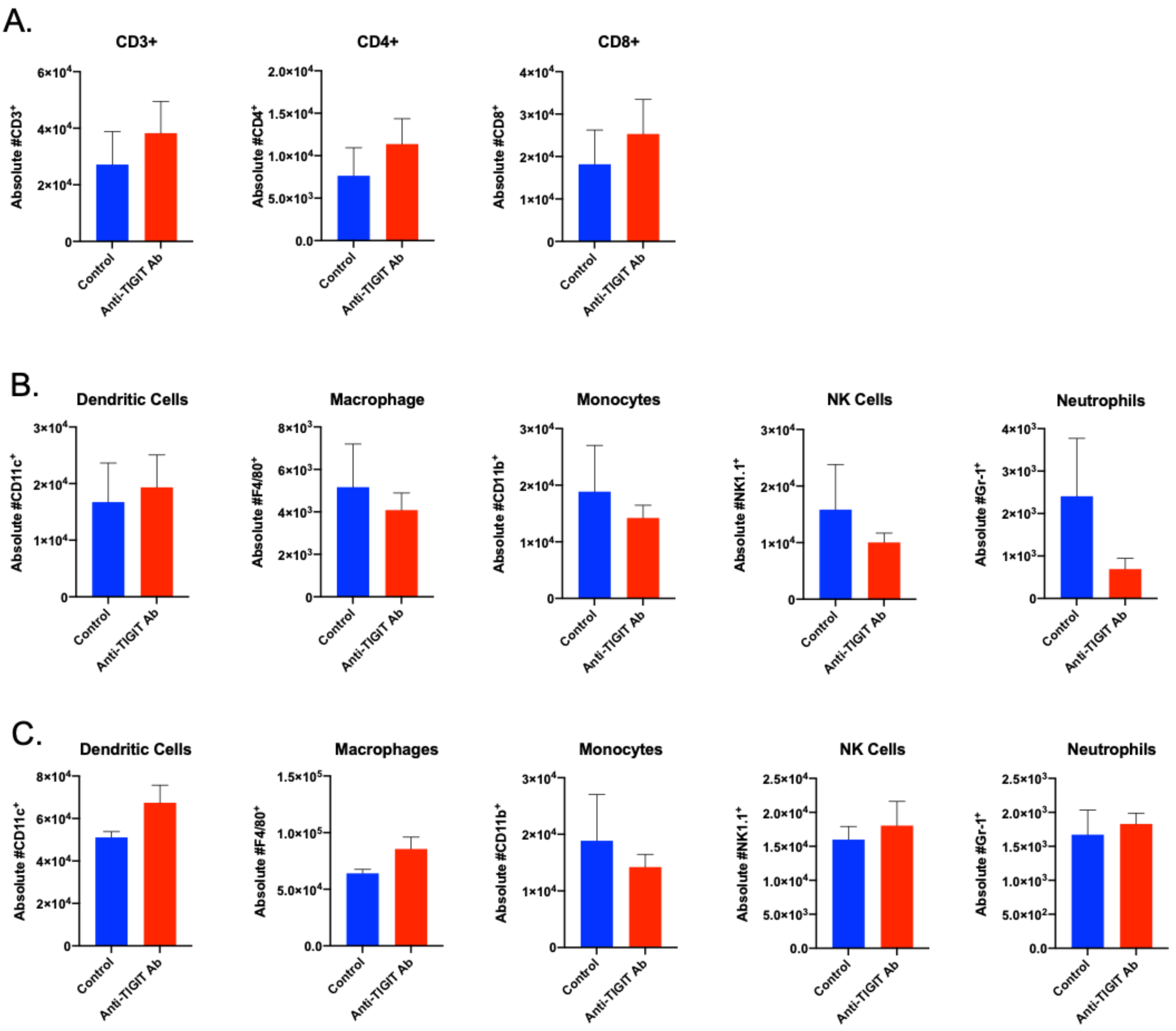

Supplemental Figure 6. Analysis of numbers of innate and adaptive immune cell subsets in the spleen and peritoneal fluid of isotype vs. anti-TIGIT treated septic memory mice. A, Peritoneal fluid was harvested from memory septic mice at 48h post CLP and numbers of CD3$^+$, CD4$^+$, and CD8$^+$ T cells were analyzed by flow cytometry. B, Numbers of CD11c$^+$ DC, F4/80$^+$ macrophages, CD11b$^+$ monocytes, NK1.1$^+$ NK cells, and Gr-1$^+$ neutrophils were assessed in the peritoneal fluid at 48h post-CLP by flow cytometry. C, , Numbers of CD11c$^+$ DC, F4/80$^+$ macrophages, CD11b$^+$ monocytes, NK1.1$^+$ NK cells, and Gr-1$^+$ neutrophils were assessed in the spleen at 48h post-CLP by flow cytometry. Groups were compared using Mann-Whitney nonparametric test.
